# Nrf1 to the rescue

**DOI:** 10.7554/eLife.02062

**Published:** 2014-01-21

**Authors:** Jin Ye

**Affiliations:** 1 **Jin Ye** is in the Department of Molecular Genetics, University of Texas Southwestern Medical Center, Dallas, United Statesjin.ye@utsouthwestern.edu

**Keywords:** p97, Nrf1, proteasomes, Human, Mouse

## Abstract

When the level of proteasomal activity in a cell drops off, a transcription factor called Nrf1 travels to the nucleus to activate the genes that code for proteasomes.

**Related research article** Radhakrishnan SK, den Besten W, Deshaeis RJ. 2014. p97-dependent retrotranslocation and proteolytic processing govern formation of active Nrf1 upon proteasome inhibition. *eLife* **3**:e01856. doi: 10.7554/eLife.01856

**Image** In the absence of proteasomes, the transcription factor Nrf1 is cleaved by an unidentified enzyme, leaving the bZip domain free to travel to the cell nucleus

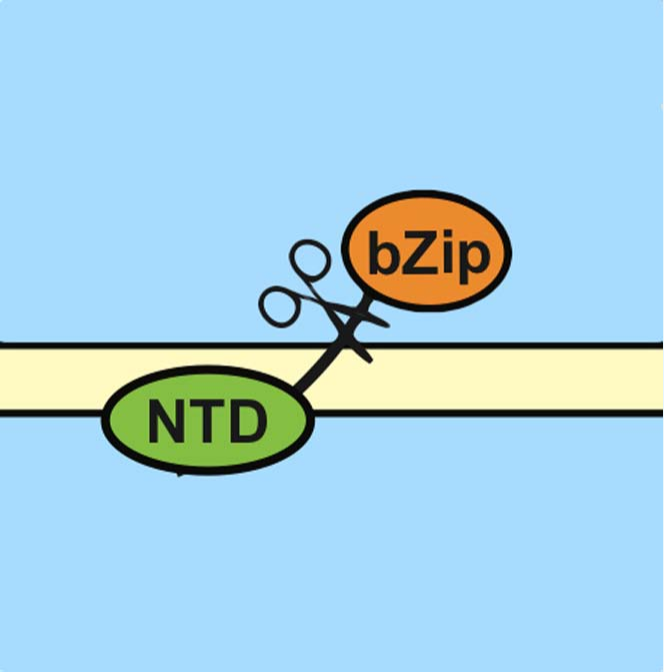

Proteasomes are protein complexes that play an important role in cells by breaking down proteins that are damaged or are not needed, thus allowing the amino acids in the proteins to be recycled to make new proteins. Sometimes the proteins that need to be broken down are in the cytosol, and sometimes they are bound to the membrane of the endoplasmic reticulum—the organelle inside which strings of amino acids are folded to make proteins. The process through which the latter proteins are broken down is known as endoplasmic reticulum-associated degradation.

Now, in *eLife*, Senthil Radhakrishnan, Willem den Besten and Raymond Deshaeis of the California Institute of Technology report that endoplasmic reticulum-associated degradation is also involved in the production of proteasomes to begin with (Radhakrishnan et al., 2014). This involvement happens via a transcription factor called Nrf1 that controls the transcription of the genes that encode the various subunits within the proteasome. The Caltech group shows that, under normal circumstances, this transcription factor is always degraded by the proteasomes. However, when the level of proteasomal activity within the cell drops, the transcription factor can travel to the nucleus of the cell and kick start production of more proteasomes—a phenomenon known as ‘bounce back’.

Endoplasmic reticulum-associated degradation begins with enzymes on this organelle adding small proteins called ubiquitins to the proteins that need to be broken down. However, the proteasomes are found in the cytosol of the cell, not the endoplasmic reticulum, so the proteins that have been ubiquitinated must somehow be brought together with the proteasomes to allow the degradation process to take place. To make this happen the parts of the proteins that are located inside the endoplasmic reticulum undergo a process called retrotranslocation—which is catalyzed by a protein called p97—that moves them to the cytosol (Vembar and Brodsky, 2008). Once the ubiquitinated proteins are exposed to the proteasomes in the cytosol, the process of breaking them down can begin.

The transcription factor Nrf1 is also produced within the endoplasmic reticulum and contains an NH_2_-terminal domain at one end and a COOH-terminal basic leucine zipper (bZip) domain at the other end (Figure 1). The bZip domain is the part of Nrf1 that activates transcription of the genes that encode the various subunits within the proteasome.

**Figure 1.**
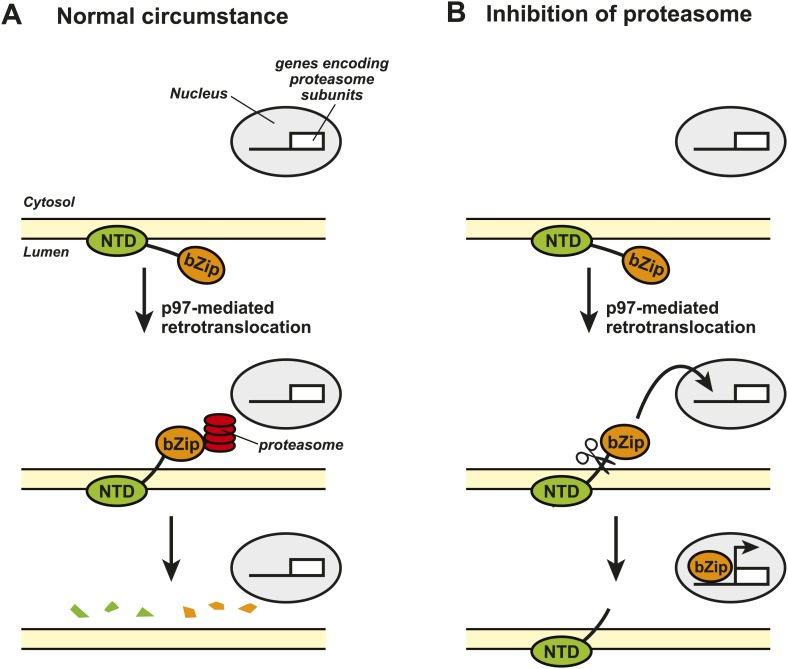
Nrf1 and the regulation of proteasome synthesis. The transcription factor Nrf1 contains an NH_2_-terminal domain (NTD) that directs it into the lumen of the endoplasmic reticulum (ER) and allows it to interact with the membrane of the ER (shown in yellow). Nrf1 also contains a COOH-terminal basic leucine zipper domain (bZip) that can activate the transcription of certain genes. (**A**) Under normal circumstances, the enzyme p97 catalyzes the retrotranslocation of Nrf1 so that the bZip domain is in the cytosol, which results in the whole protein being rapidly degraded by proteasomes. (**B**) When proteasome inhibitors are added to the cell, the retrotranslocated Nrf1 is cleaved by an unidentified enzyme. This allows the bZip domain to leave the membrane and enter the nucleus, where it activates transcription of the genes that encode the various subunits within the proteasome.

Figure credit: Nancy Heard and Jin Ye

The Caltech group showed that, under normal conditions, the bZip domain of the protein was retrotranslocated from the endoplasmic reticulum to the cytosol, where it was rapidly degraded by the proteasomes. This process happened continuously, which meant that the bZip domain never reached the target genes in the nucleus of the cell (Figure 1A). However, when the cells were treated with a proteasome inhibitor, the amount of retrotranslocated Nrf1 (and hence the amount of bZip in the cytosol) increased, which allowed an unidentified enzyme to cleave the transcription factor between the NH_2_-terminal domain and the bZip domain. Since the bZip domain was no longer attached to the membrane of the endoplasmic reticulum, it was able to enter the nucleus and activate transcription of genes encoding the proteasome subunits (Figure 1B). This mechanism allows cells to sense any reduction in the activity of proteasomes and to compensate for this by increasing the synthesis of new proteasomes.

The work of the Caltech group is significant as it reveals the signaling mechanism that regulates proteasome synthesis. This knowledge should be useful in developing novel strategies to treat diseases in which abnormal proteasomal activity is involved. For example, neurodegenerative diseases such as Parkinson’s disease and various Prion diseases are characterized by the accumulation of ubiquitinated protein aggregates in neurons, which suggests that there too little proteasomal activity within these cells (Lehman, 2009). Is the expression or activation of Nrf1 impaired in these neurons? If so, will activation of the Nrf1 signaling pathway help to eliminate these aggregates? Future studies to address these questions may yield desperately needed novel strategies to combat these diseases by increasing proteasome synthesis.

Treating certain cancers, on the other hand, will require the opposite approach. Proteasome inhibitors have been used to treat multiple myeloma by blocking the degradation of proteins that inhibit the proliferation of cancer cells (Mahindra et al., 2012). Although this treatment is effective at first, nearly all patients eventually relapse because the increased expression of proteasome subunits in the cancer cells leads to drug resistance. Therefore, inhibiting the activation of Nrf1 might make such treatment more effective. This could be done by targeting p97, the protein that catalyzes the retrotranslocation process: however, p97 performs multiple functions inside cells (Halawani and Latterich, 2006), so a better target would be the unidentified enzyme that cleaves Nrf1. Identification of this enzyme is, therefore, a clear priority for future research.
